# Lupinine as an Acetylcholinesterase Inhibitor from *Anabasis salsa* (C.A. Mey.) Benth. ex Volkens—Isolation by Centrifugal Partition Chromatography

**DOI:** 10.3390/molecules31142452

**Published:** 2026-07-13

**Authors:** Pernesh Zh. Bekisheva, Malgorzata Kozyra, Maryna Koval, Khorlan I. Itzhanova, Zhangeldy S. Nurmaganbetov, Wirginia Kukula-Koch

**Affiliations:** 1 School of Pharmacy, NCJSC «Karaganda Medical University», Gogol Str. 40, Karaganda 100008, Kazakhstan; pernesh1983@mail.ru (P.Z.B.); itzhanova65@mail.ru (K.I.I.); 2 Department of Pharmacognosy with Medicinal Plants Garden, Medical University of Lublin, 1 Chodzki Str., 20-093 Lublin, Poland; malgorzata.kozyra@umlub.edu.pl (M.K.); marinchik.koval@gmail.com (M.K.); 3 Doctoral School of the Medical University of Lublin, Medical University of Lublin, 7 Chodzki Str., 20-093 Lublin, Poland

**Keywords:** *Anabasis salsa*, alkaloids, lupinine, centrifugal partition chromatography, acetylcholinesterase activity

## Abstract

This study investigates the phytochemical composition and acetylcholinesterase (AChE) inhibitory activity of *Anabasis salsa* (C.A. Mey.) Benth. ex Volkens (Chenopodiaceae), with a particular focus on secondary metabolites present in its extracts. The plant extracts were profiled using HPLC-ESI-QTOF-MS/MS, revealing a diverse composition including organic acids, phenolic compounds, flavonoids, and alkaloids. The latter were represented by anabasine and lupinine that were identified as significantly present compounds. Depending on the type of extract and plant part, lupinine was present in the extracts at a quantity range from 0.0033 to 6.35%, and its highest concentration was noted in the acidified macerate from the overground parts of the plant. Centrifugal partition chromatography (CPC) operated using the solvent system composed of MTBE:ACN:n-BuOH:H_2_O, 2:2:1:5 (*v*/*v*/*v*/*v*), was applied for the fractionation of the most promising extract, enabling efficient separation using a biphasic solvent system. The obtained fractions were evaluated for AChE inhibitory activity, and the most active fractions were found to contain lupinine, which was successfully isolated in a purified fraction using CPC. These findings highlight lupinine as a significant bioactive compound with neuroprotective potential and demonstrate the applicability of CPC as an efficient and scalable method for the isolation of alkaloids from complex plant matrices.

## 1. Introduction

Kazakhstan is characterised by a rich diversity of medicinal plant resources, many of which are suitable for large-scale industrial utilisation. The growing demand for highly effective and low-toxicity herbal preparations has been substantiated by extensive studies on biologically active compounds and the development of plant-derived pharmaceuticals. This progress has contributed to reducing the dependence of the national healthcare system on imported drugs. Addressing this challenge requires the effective use of domestic raw materials, production capacity, and technological potential. In this context, the search for new sources of biologically active compounds and the development of efficient technologies for their extraction and processing remain highly relevant. Notably, plant-derived drugs occupy a dominant position among medicinal products, with approximately 30% containing alkaloids or their functional nitrogen-containing derivatives [1].

The genus *Anabasis* (family Chenopodiaceae) is one of the most prominent genera within this family, comprising 29 species distributed across tropical, temperate, saline, arid, and semi-arid regions worldwide [2]. Various species of this genus are widely used in traditional medicine. For example, *A. articulata* is employed in Algeria for the treatment of skin diseases, diabetes, fever, and headaches [3,4], while *A. syriaca* is used in Jordan to regulate childbirth and menstruation, stimulate the respiratory system, and treat liver diseases and ulcers [5]. Additionally, *A. aphylla* has been applied as a botanical insecticide in northwest China [6].

Previous biological studies have demonstrated that *Anabasis* species exhibit antioxidant, antibacterial, and antidiabetic activities, primarily attributed to their rich content of secondary metabolites [2,4,7]. Phytochemical investigations have revealed the presence of diverse compounds, including isoflavonoids and glucosidic derivatives isolated from *A. salsa* and *A. brevifolia* [8]. Furthermore, compounds such as picraquassioside C, syringin, piceoside, tortoside A, and phytolaccagenic acid derivatives were isolated for the first time from the n-butanol extract of *A. salsa* [9]. Other studies reported the presence of triterpenoids and triterpene saponins in different *Anabasis* species [10,11], as well as a wide range of secondary metabolites including saponins, alkaloids, phenolics, terpenes, and sterols [12,13].

Among the less-studied alkaloid-containing species is *Anabasis salsa* (C.A. Mey.) Benth. ex Volkens., a halophytic perennial subshrub native to the steppe and semi-desert regions of Kazakhstan and Central Asia. This species is widely distributed across Western, Southern, and Central Kazakhstan, typically growing on saline clay soils or stony loams. Its aboveground biomass yield ranges from 80 to 395 kg/ha, with total exploitable reserves estimated at 3099.87 tons and an annual harvest potential of 1852.89 tons [14]. Phytochemical studies have identified various classes of biologically active compounds in A. salsa, including alkaloids, saponins, flavonoids, coumarins, phenols, glycosides, carbohydrates, and vitamins C and B2, which are associated with diverse pharmacological activities [9].

*Anabasis salsa* (C.A. Mey.) Benth. ex Volkens (*A. salsa*) is widely used in traditional medicine. Aqueous infusions of its seeds are applied in the treatment of paralysis and neuritis, while alcoholic extracts are used for muscle atrophy and psoriasis. Root infusions have been reported to exhibit antihypertensive effects, and dried flower heads are used for trigeminal nerve inflammation. The plant has also been used in the management of sexual dysfunction, hysteria, physical and mental exhaustion, atherosclerosis and central nervous system disorders, proving its ability to cross the blood–brain barrier [15].

Previous investigations have included morphological and histochemical studies of *A. salsa* growing in Central Kazakhstan, which identified key diagnostic features and confirmed the presence and localisation of phenolic compounds, flavonoids, and alkaloids in various plant tissues [16]. Despite these findings, the phytochemical composition of this species, particularly its alkaloid fraction, remains insufficiently explored. In this case, alkaloids are of particular interest due to their pronounced biological activity. Numerous studies have demonstrated their pharmacological effects, including actions on the circulatory system, metabolic regulation (including anti-obesity effects), and treatment of cardiovascular and skin disorders [17]. In addition, alkaloids exhibit hypoglycemic, hypolipidemic, antimicrobial, antiviral, anti-hepatitis, anti-inflammatory, antioxidant, anticancer, and neuroprotective properties, as well as sedative effects on the central nervous system [18,19]. Among them, quinolizidine alkaloids such as anabasine and lupinine are especially noteworthy, as they are abundant in wild-growing *Anabasis* species in Kazakhstan.

Lupinine, a major alkaloid of the genus *Anabasis*, exhibits bactericidal, mild sedative, anthelmintic, and hypotensive activities, making it a compound of considerable pharmacological interest. Although not used directly in its native form, lupinine serves as a precursor for the synthesis of lupicaine hydrochloride, a local anaesthetic. Furthermore, its ester derivatives have demonstrated antiviral and hepatoprotective properties [20,21,22]. Previous studies have also reported a variety of structurally modified lupinine derivatives with significant biological activity [23,24,25,26,27].

Despite the recognised pharmacological potential of *A. salsa*, comprehensive studies focusing on its alkaloid profile and bioactivity remain limited. Therefore, the aim of the present study was to characterise the chemical composition of *A. salsa* extract and to fractionate it by centrifugal partition chromatography (CPC) as a modern and solvent-efficient technique, and to isolate lupinine for testing of its acetylcholinesterase inhibitory potential, providing insight into their potential neuroprotective applications.

The inhibition of acetylcholinesterase (AChE) represents a key therapeutic strategy in the management of neurodegenerative disorders, particularly Alzheimer’s disease (AD). AChE is responsible for the rapid hydrolysis of acetylcholine, a neurotransmitter essential for cognitive processes such as learning and memory, and its excessive activity contributes to cholinergic dysfunction observed in AD. According to the cholinergic hypothesis, the degeneration of cholinergic neurons and the resulting decrease in acetylcholine levels are directly associated with cognitive decline in affected patients. Although currently available drugs, such as donepezil, rivastigmine, galantamine, and memantine, provide symptomatic relief, they do not halt disease progression and are often associated with adverse effects and reduced efficacy during long-term treatment [28].

These limitations highlight the urgent need to discover novel, safe, and more effective AChE inhibitors. In this context, natural products, particularly plant-derived compounds, remain a promising source of new therapeutic candidates due to their structural diversity and broad spectrum of biological activities. With the application of green and scalable techniques, such as CPC, the recovery of active compounds on an industrial scale is becoming possible in recent years.

## 2. Results and Discussion

### 2.1. The Extract Profiling by HPLC-ESI-Q-TOF-MS/MS and Semi-Quantitative Analysis of the Extracts’ Composition

**Figure 1:**
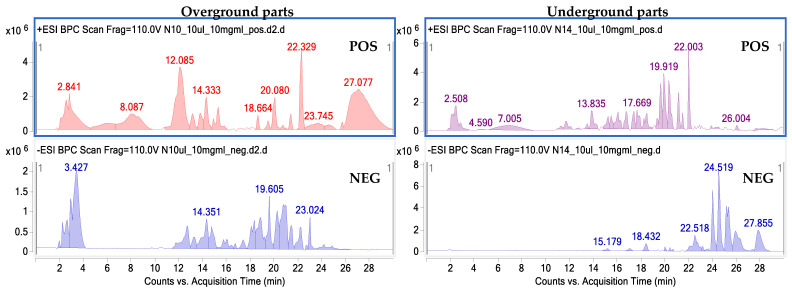
Total ion chromatograms (TICs) of *Anabasis salsa* overground and underground parts in positive ion (POS) and negative ion (NEG) modes.

The fingerprints of the extracts were obtained by the HPLC-ESI-QTOF-MS/MS analysis. The applied gradient of solvents led to the separation of metabolites present in the tested samples. In total, different secondary metabolites were tentatively identified in various organs of the plant, based on the comparison with reference compounds, the analysis of scientific literature and the studies on their mechanism of fragmentation. Clear fingerprints, recorded for both the aboveground and underground parts in the positive and negative ion modes, were obtained (Figure 1), and the compounds’ assignment is presented in Table 1, and they resembled the characteristics described in the scientific literature [2,29,30,31,32,33,34,35,36].

Based on accurate mass measurements, literature data, and analysis of fragmentation patterns, several metabolites were tentatively assigned as components of the extracts. The table below contains the list of proposed compounds, and Table S1 in the Supplementary File includes their MS/MS fragmentation spectra.

The HPLC-ESI-QTOF-MS/MS profiling of *Anabasis salsa* extracts revealed a diverse and complex composition of secondary metabolites from several classes. Among the identified constituents, organic acids were well represented, including citric acid, malic acid, succinic acid, and quinic acid. These primary metabolites may contribute to the physicochemical properties of the extracts and influence the extraction efficiency of other compounds. A significant group of compounds consisted of phenolic acids and their derivatives, such as protocatechuic acid, caffeic acid, coumaric acid, ferulic acid, vanillic acid, and syringic acid, along with their glycosylated forms (e.g., syringic acid hexoside and vanillic acid hexoside). The presence of these metabolites indicates an active phenylpropanoid pathway and suggests potential antioxidant properties of the extracts.

Additionally, tannin-related derivatives were identified, including galloylglucose, which represents a typical ester of gallic acid and glucose. The extracts were also rich in flavonoids, detected both as aglycones and glycosides. Identified compounds included luteolin glucoside (orientin), quercetin dirhamnoside (rutoside), and isorhamnetin hexoside, confirming a high abundance of polyphenolic constituents in the studied material. 

Particularly interesting was the presence of two alkaloids, detected in the positive ionisation mode. Two major compounds were identified: anabasine and lupinine. Anabasine was detected in extracts AS-70P (No. 9), AS-90P (No. 11), AS-90t (No. 13), and AS-K-90 (No. 14), whereas lupinine was observed in a broader range of samples, including AS-70P (No. 9) and ethanolic extracts obtained by maceration and percolation (No. 12–15).

The MS/MS fragmentation patterns of these alkaloids were characteristic. Anabasine (*m*/*z* 163) showed fragment ions at *m*/*z* 146, 134, and 120, corresponding to sequential losses of alkyl fragments and cleavage of the pyridine ring. In contrast, lupinine (*m*/*z* 170) exhibited fragment ions at *m*/*z* 152, 137, and 124, reflecting the fragmentation of the quinolizidine skeleton and elimination of small neutral molecules. 

Both alkaloids are well known for their biological activity. Anabasine interacts with nicotinic acetylcholine receptors [37], while lupinine, a quinolizidine alkaloid, has documented pharmacological properties, including potential neuroprotective effects and acetylcholinesterase inhibitory activity [38]. This chemical diversity supports their significant biological potential and justifies further investigation, particularly in the context of acetylcholinesterase inhibition.

Metabolite annotation proved particularly challenging in the positive ESI mode. The majority of detected ions could not be confidently identified owing to the limited availability of reference MS/MS spectra and the scarcity of phytochemical data concerning the alkaloid composition of *Anabasis* species and the family Chenopodiaceae. In addition to the previously identified major alkaloids, additional nitrogen-containing metabolites were tentatively characterised using accurate mass measurements, elemental composition, MS/MS fragmentation patterns, and chemotaxonomic considerations.

Two metabolites with the molecular formulas C_18_H_20_N_2_O_2_ and C_18_H_20_N_2_O_3_ were tentatively assigned to the quinolizidine alkaloid class (Schymanski level 3). Their high-resolution MS/MS spectra exhibited characteristic neutral losses consistent with oxygenated quinolizidine-type skeletons, including sequential losses of small neutral fragments such as H_2_O and CO, together with cleavage of the heterocyclic framework, producing abundant low-mass nitrogen-containing fragment ions. Although these fragmentation patterns are compatible with quinolizidine alkaloids, no unequivocal match with authentic standards or reference spectra was obtained. Furthermore, quinolizidine alkaloids have not previously been reported from Anabasis species. Therefore, these metabolites are conservatively assigned only to the quinolizidine alkaloid class, and their structural identification should be regarded as tentative.

An additional metabolite (*m*/*z* 232.1332, C_14_H_17_NO_2_) was included in the phytochemical profile as a pyridine alkaloid derivative. This assignment was supported by its elemental composition and positive-ion MS/MS fragmentation, which generated characteristic nitrogen-containing fragment ions consistent with a substituted pyridine-containing skeleton. However, the available spectral evidence was insufficient for proposing a specific molecular structure; therefore, only the chemical class was assigned.

Based on previous phytochemical investigations of the genus, yaksartinine (*m*/*z* 166.1226, C_10_H_15_NO) was also included in the metabolite table. The detected precursor ion produced major fragment ions at *m*/*z* 121.0646, 103.0540, and 93.0697, which are consistent with previously reported fragmentation of this alkaloid. Because yaksartinine has already been described in *Anabasis* species, its occurrence in the present extract is supported by both the LC–HRMS/MS data and published chemotaxonomic evidence.

*Anabasis* genus has received the attention of the scientific community because of its characteristic biologically active secondary metabolites. To date, only a few scientific works have described the composition of *Anabasis* species extracts.

*A. salsa* has been an underestimated and insufficiently investigated plant species. For centuries, it has been perceived as an alkaloid-containing plant, whereas the outcomes of current research clearly show plenty of secondary metabolites of different kinds that are present mainly in its leaves and stems. Previously, other authors [39] examined the aerial parts of *A. salsa*, which were collected in the South Kazakhstan region, near the Syrdarya River in March 2022. Petroleum ether and dichloromethane extracts were subjected to GC–MS analysis, and valuable bioactive compounds were identified from the chromatograms. Additionally, butanol extract was subjected to HPLC–UV analysis, and the presence of gallic acid was confirmed. Overall, results confirmed the presence of 108 various compounds from different chemical classes: fatty acids and derivatives, steroidal compounds, phenolic compounds and minor group compounds.

Thus, five glucosidic and isoflavonoid compounds were isolated from butanol-extract of *A. salsa* and chloroform-extract of *A. brevifolia*, such as 2-O-β-Dglucopyranosyloxy-4,6-dimethoxy phenylenthanone, 5,6,7,2′-tetramethoxy isoflavonoid, 2-O-(2)-β-D-glucopyranosyloxy-4, 6-dimethoxy phenylenthanone, 3-methyl-but-2-enoic acid-[2-(4-methoxy phenyl)-ethyl]-amide and 2′-hydroxy-5,6,7-trimethoxyisoflavonoid [40]. In addition, five new molecules were isolated and identified in the n-BuOH extract of *A. salsa* (tortoside A, phytolaccagenicacid-3-O-β-D-glucopyranuronide 28-βD-glucopyranosyl ester, picraquassioside C, syringin and piceoside) [15].

In *A. setifera*, oleanolic and 2-α-hydroxy ursolic acids were the most abundant sapogenins that were isolated in this species. They were usually detected as glycosylated with rhamnose or galactose [11]. Finally, jaxartinine was another alkaloid isolated from *A. jaxartica* [41]. This study presents a GC-MS procedure which was applied to identify alkaloids in *A. articulata* stems (Algerian origin). According to the literature, 49 compounds divided into 16 families, namely cyclopeptide alkaloids, steroid alkaloids, quinoline alkaloids, camptothecin alkaloids, quinazoline alkaloids, isoquinoline alkaloids, isoquinolone alkaloids, indole alkaloids, terpene indole alkaloids, pyridine/pyrrolidine alkaloids, piperidine alkaloids, pyrrolizidine alkaloids, purine alkaloids, acridone alkaloids, benzazonine alkaloids and homolycorine-type Amaryllidaceae alkaloids, were identified. Therefore, *A. articulata* can be considered a source of antioxidant and antimicrobial agents [2].

Then, several types of alkaloids from *A. aphylla*, like anabasine (neonicotine), anabasamine, lupinine, aphylline, aphylline N-oxide, aphyllidine, oxaphylline, lupinine methylanaphyllinate N-methylanabasine and isonicoteine, were isolated and characterised in other studies [42,43]. As is already known, in the early stages of development in *A. aphylla*, accumulation occurs in the above-ground part—the content of alkaloids is 2.7 times higher than in the underground organs of the plant. In later stages of development, the alkaloid accumulates in all organs, especially in the stems. *A. aphylla’s* maximum alkaloid content is reached in summer—2.36%, in autumn—14.14%. *A. aphylla* alkaloids are quite widespread in the plant world. In *A. aphylla,* they are localised mainly in the aboveground part (0.54–1.095%), and there are fewer of them in the roots (0.17–0.26%). This distribution can be explained by the special role of the alkaloid anabasine, which is involved in the redox processes of anabasis metabolism. The accumulation of anabasine occurs both during the flowering period and in the later stages of development, because it takes an active part in nitrogen metabolism: its content in summer is 1.26%, in autumn—1.44% in total [44]. In this study, the authors confirmed the findings of other research groups that focused on the determination of alkaloids’ composition in *Anabasis* L. [8,30,31,45,46,47,48,49,50,51]. Similarly to the other results, lupinine and anabasin were identified in the investigated samples (Table 1).

In view of quantitative data previously reported for *Anabasis* species, an approximate estimation of lupinine content in the tested extracts was performed to support the selection of samples for further CPC fractionation. For this purpose, a reference standard of lupinine was analysed at several concentrations, and a direct comparison of peak areas between the standard solutions and the examined extracts was carried out using HPLC-MS. Based on this approach, the approximate percentage content of lupinine in the extracts was estimated (Table 2). It should be noted that these values are approximate, as the method was not fully validated; however, they provide a reliable overview of the order of magnitude and relative differences in lupinine content among the investigated samples.

Lupinine was detected in all extracts obtained from both aerial parts (stems, leaves, and flowers) and roots of *A. salsa*. However, significant differences in its content were observed depending on the extraction conditions. The lowest amounts of lupinine were found in chloroform extracts (AS-X-t, extract 15: 0.0179%; AS-K-x, extract 16: 0.0080%), indicating that this solvent system is not efficient for the extraction of this polar alkaloid.

In contrast, the highest lupinine content was observed in the 90% ethanolic extract obtained after alkalisation of the plant material (AS-90tP, extract 12: 6.3523%), which markedly exceeded the levels detected in all other extracts. This result clearly demonstrates the crucial role of alkalisation in enhancing the extraction efficiency of alkaloids. Other ethanolic extracts showed moderate or low lupinine content, including AS-90t (extract 13: 0.5554%) and AS-90P (extract 11: 0.0202%), while the 70% ethanolic extracts contained only trace amounts (AS-70P, extract 9: 0.0727%; AS-70t, extract 10: 0.0033%). The root extract obtained with 90% ethanol (AS-K-90, extract 14: 0.0265%) also exhibited relatively low levels of lupinine. Still, the overground parts of the plant were better sources of lupinine. Based on these results, the extract with the highest alkaloid content, namely the alkalised ethanolic extract (extract 12), was selected for further fractionation using CPC.

### 2.2. CPC Fractionation of the Anabasis salsa (C.A. Mey.) Benth. ex Volkens Extract

Centrifugal partition chromatography (CPC), also known as support-free liquid–liquid chromatography (SFC/SVC), has emerged as a rapidly developing technique for industrial-scale isolation of individual plant-derived metabolites from complex mixtures. In contrast to conventional chromatographic methods based on solid stationary phases, CPC operates using a biphasic system composed of two immiscible liquid phases [52].

In this system, one liquid phase is retained in the column as the stationary phase, while the other is continuously pumped through the column as the mobile phase. The column itself is subjected to a centrifugal field generated by rotation, which enables the retention of the stationary phase within a series of interconnected chambers. Upon introduction of the sample, its components are distributed between the two liquid phases according to their partition coefficients, resulting in separation based on differential affinity toward the upper or lower phase [53].

The absence of a solid support eliminates irreversible adsorption phenomena, significantly reducing sample loss and improving recovery efficiency. This feature is particularly advantageous in the isolation of sensitive natural products, including alkaloids, which are prone to degradation or strong interactions with solid matrices.

Another important advantage of CPC is the flexibility in selecting solvent systems. The use of various combinations of immiscible solvents allows for fine-tuning of the separation conditions and enhances selectivity toward specific classes of compounds, depending on their physicochemical properties. Moreover, the process is typically conducted under mild temperature conditions, which further supports the stability of thermolabile constituents [54].

CPC is also characterised by high reproducibility and scalability. The separation conditions can be readily transferred from laboratory to industrial scale by adjusting the volume ratios of the solvent system relative to the column capacity, making this technique highly attractive for preparative and semi-preparative applications.

Due to these advantages, CPC has found increasing applications in the pharmaceutical, nutraceutical, and food industries. It has been successfully employed, for example, in the purification of cannabinoids such as CBD, the separation of omega fatty acids, and the isolation of polyphenolic complexes such as catechins from green tea [39,53,55].

In the present study, CPC was applied for the fractionation of *Anabasis salsa* extracts and for the screening of fractions with respect to their acetylcholinesterase (AChE) inhibitory activity. AChE is a key enzyme involved in the hydrolysis of acetylcholine, and its excessive activity is associated with cognitive decline and memory impairment during ageing. Therefore, the identification of natural inhibitors of this enzyme remains an important objective in the search for compounds with potential neuroprotective properties.

Previous reports describe the isolation of lupinine using multistep procedures involving alkalization of the plant material, repeated acid–base liquid–liquid extraction, chromatographic purification on silica gel or alumina, TLC monitoring of individual fractions, and, in some cases, final recrystallization of the isolated alkaloid Alternative methods described in earlier studies include chemical derivatisation strategies, such as acetylation of total alkaloids to form O-acetyl derivatives, followed by fractionation and hydrolysis to recover pure lupinine in high yields. While effective, these approaches are often labour-intensive, involve the use of large amounts of reagents, and may require harsh chemical conditions or multiple processing stages [56,57]. In contrast, CPC is a support-free liquid–liquid chromatographic technique that does not require a solid stationary phase, thereby eliminating irreversible adsorption of analytes and reducing sample losses. Furthermore, the entire purification is performed in a single chromatographic run, avoiding repeated column chromatography and substantially reducing solvent consumption associated with multiple purification steps [58]. An additional advantage of CPC is its straightforward scalability, as preparative conditions can be transferred directly to larger column volumes without changing the separation mechanism. In this context, the application of CPC offers a promising alternative for the enrichment of lupinine. 

#### 2.2.1. The Selection of the Biphasic Solvent Composition for the Fractionation of *Anabasis salsa* (C.A. Mey.) Benth. ex Volkens Extracts

The selection of a biphasic solvent system that ensures efficient and selective fractionation of complex plant extracts is a crucial step in centrifugal partition chromatography (CPC) [53,59]. In the present study, aimed at the isolation of alkaloids from *Anabasis salsa* hydroalcoholic extract (90% EtOH), several biphasic solvent systems were evaluated to determine the most suitable conditions for CPC separation.

A total of six solvent systems (Table S2, Supplementary File) were tested, differing in polarity and composition, including combinations of n-hexane, ethyl acetate, ethanol, n-butanol, methyl tert-butyl ether (MTBE), acetonitrile, and aqueous phases. The selection process was based on the determination of partition coefficients (K values) for the major constituents of the extract (Table S3), calculated as the ratio of compound concentration in the stationary phase to that in the mobile phase.

An optimal CPC solvent system should provide K values within the range of 0.5–2.0, ensuring sufficient retention of analytes in the stationary phase while allowing their efficient elution. Systems yielding K values significantly lower than 0.5 result in rapid elution and poor resolution, whereas values above 2.0 lead to excessive retention times and peak broadening [59].

Among the tested systems, solvent system No. 4 (MTBE:ACN:n-BuOH:H_2_O, 2:2:1:5, *v*/*v*/*v*/*v*) demonstrated the most favourable partition behaviour. As shown in Table S3, the investigated compounds were effectively distributed between the two immiscible phases, with several major constituents exhibiting K values below unity, while others showed K values above 1. This balanced distribution indicates adequate selectivity and suggests that the system is suitable for the separation of structurally diverse metabolites present in the extract.

Importantly, the K values obtained for the key compounds in system No. 4 were within or close to the optimal range (0.5–2.0), indicating appropriate retention and elution profiles during CPC operation. This behaviour is particularly advantageous for complex mixtures, as it allows prolonged interaction of analytes with the stationary phase, resulting in improved resolution and fractionation efficiency.

Another important factor influencing the selection of the solvent system was its composition. Unlike some of the tested systems, system No. 4 did not require the addition of acidic or basic modifiers. The absence of additives such as hydrochloric acid or triethylamine simplifies downstream processing and facilitates solvent removal, which is especially relevant in the context of potential scale-up and technological applications.

Based on the obtained results, solvent system No. 4 was selected for further CPC fractionation of the *A. salsa* extract. This system provided a suitable balance between selectivity, efficiency, and practicality, making it a promising candidate for the isolation of alkaloid constituents.

#### 2.2.2. The CPC Fractionation of the *Anabasis salsa* Extract

**Figure 2:**
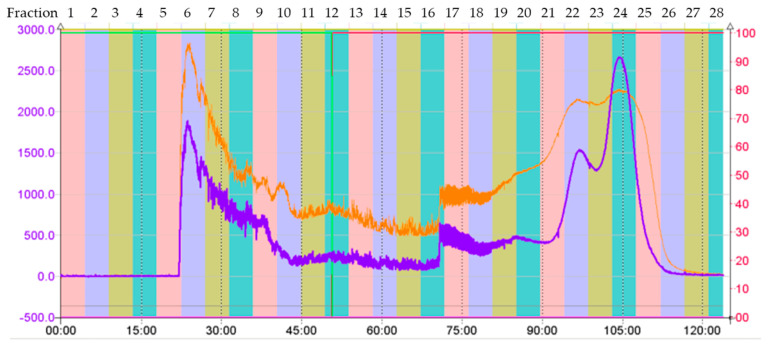
The CPC chromatogram obtained for the *Anabasis salsa* extract using the biphasic solvent system composed of MtBE-ACN-BuOH-H_2_O 2:2:1:5 (*v*/*v*/*v*/*v*) in the ascending mode, with lupinine in fraction 24.

The CPC separation of the *Anabasis salsa* extract was performed using the biphasic solvent system composed of MTBE: acetonitrile: n-butanol: water (2:2:1:5, *v*/*v*/*v*/*v*), previously selected based on partition coefficient evaluation. The CPC column was first filled with the stationary phase (lower phase), after which the sample (600 mg) was introduced into the system. The mobile phase was then pumped through the column under controlled conditions. The separation was carried out at a rotational speed of 1200 rpm. After approximately 20 min of operation, the first peaks appeared on the CPC chromatogram, indicating the elution of less retained compounds. During the elution step, a total of 12 fractions were collected. Following this stage, the separation was continued in the extrusion mode until 120 min, allowing for the recovery of more strongly retained compounds. In total, 28 fractions were collected throughout the entire process. The flow rate during the elution mode was maintained at 4 mL/min, and in the extrusion mode—at 6 mL/min. Under these conditions, efficient fractionation of the extract was achieved, resulting in well-resolved chromatographic profiles (Figure 2).

Subsequently, all collected fractions were subjected to compositional analysis. Based on their chemical profiles, the fractions were grouped according to the presence of similar metabolites and directed to the acetylcholinesterase inhibition activity assay. 

### 2.3. Acetylcholinesterase Inhibitory Activity

**Figure 3:**
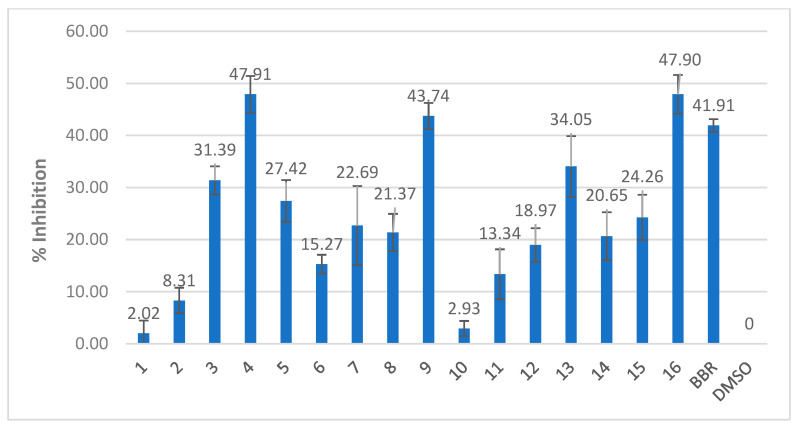
Percentage of acetylcholinesterase (AChE) inhibitory activity of the studied samples and berberine chloride (BBR) as a reference standard determined by a fluorometric assay. Results are presented as the mean ± standard deviation (SD) of three technical replicates (n = 3) and expressed as percentage inhibition (%). Sample numbering: 1 = 6–7, 2 = 8–10, 3 = 12–16, 4 = 17–18, 5 = 19–20, 6 = 21, 7 = 22, 8 = 23, 9 = 24–25, 10 = AS-water K (17), 11 = AS-water H (18), 12 = AS-70P (9), 13 = AS-90tP (12), 14 = AS-X-t (15), 15 = AS-K-x (16), 16 = AS-Lup (lupinine standard).

The AChE inhibitory activity of the studied crude extracts and CPC fractions is presented in Figure 3. Since lupinine was identified in both the crude extracts and the CPC fractions, the lupinine standard was also evaluated to compare its inhibitory potential with that of the tested samples.

Fractions 4 (17–18) and 9 (24) exhibited the highest acetylcholinesterase (AChE) inhibitory activity, with inhibition values of 47.91 ± 3.53% and 43.74 ± 2.50%, respectively. Among the standards, AS-Lup (16) showed the highest inhibitory activity (47.90 ± 3.70%), followed by berberine chloride (BBR) (41.91 ± 1.25%).

The compositional analysis of these fractions (See Table S4, Supplementary File) indicated the presence of lupinine. The high AChE inhibitory activity observed for both the lupinine-enriched CPC fraction and the lupinine standard suggests that this alkaloid contributes to the observed biological activity. However, the lack of a direct correlation between lupinine content and the activity of crude extracts (12 AS-70P) indicates that additional constituents and/or synergistic interactions may also influence the overall inhibitory effect.

In particular, fraction 9 was found to be enriched in lupinine, whereas fraction 4 contained a dominant single peak, for which the MS/MS spectrum confirmed the presence of a characteristic fragment ion typical for anabasine or a structurally related isomer (See Figure S1, Supplementary File).

Interestingly, the detected ion at *m*/*z* 136 in sample 4 may correspond to an in-source fragment of anabasine-type alkaloids rather than an individual compound. The MS/MS spectrum of this ion showed a dominant fragment at *m*/*z* 105, resulting from a neutral loss of 31 Da (methylamine), which is characteristic for alkaloids containing aliphatic amine moieties. Therefore, further structural elucidation of this compound would require the application of additional analytical techniques, such as nuclear magnetic resonance (NMR) spectroscopy, to confirm its exact structure.

Taking into account that lupinine was consistently present in the most active fractions, its role as a major bioactive constituent was further investigated using a reference standard. As demonstrated in Figure 3, lupinine (sample 16) exhibited stronger inhibitory activity than berberine, a well-known acetylcholinesterase inhibitor, under the applied experimental conditions.

These findings indicate that lupinine is a significant contributor to the AChE inhibitory activity of *Anabasis salsa* extracts. Given the established role of acetylcholinesterase inhibition in the symptomatic management of neurodegenerative disorders, these results suggest that lupinine may be a promising lead compound for further studies of neuroprotective agents. However, neuroprotective activity was not directly evaluated in the present study. Previous reports have demonstrated that quinolizidine alkaloids and structurally modified lupinine derivatives exhibit neurotropic and analgesic activities, supporting further investigation of this scaffold [60,61].

Lupinine is a lipophilic pharmacophore that, through its actions on serotonin receptors and cholinesterase inhibition, can directly influence the central nervous system. Due to the high lipophilicity of the quinolizidine core, the lupinine molecule and its esters effectively cross the blood–brain barrier, providing a direct, targeted effect on brain synapses. Modifications of the lupinine octahydroquinolizidine core enable the creation of effective drugs with proven antimalarial, antituberculosis, antifungal, and neurotropic activity [40,60,61].

## 3. Materials and Methods

### 3.1. Plant Material

**Figure 4:**
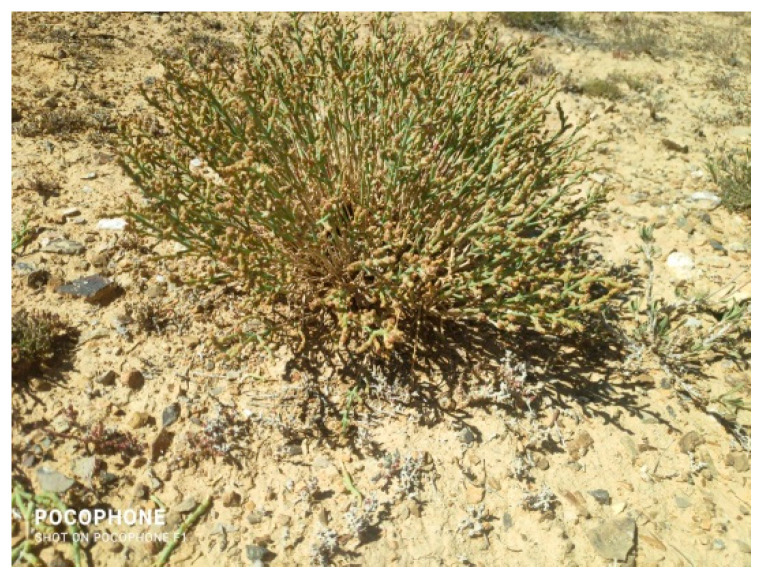
Appearance of *Anabasis salsa* (C.A. Mey.) Benth. ex Volkens (flowering phase).

Aerial and underground parts of *A. salsa* (Figure 4) were collected at the flowering stage in the vicinity of Akzhal village (Shetskiy District, Karaganda Region, Republic of Kazakhstan) between August and September 2023. The plant material was taxonomically identified by specialists from the Department of Botany, Karaganda Buketov University. A voucher specimen was deposited in the herbarium of the Faculty of Biology and Geography of the same institution. The collected material was cleaned to remove impurities, air-dried in the shade at ambient temperature to constant weight, and ground into a fine powder prior to extraction.

### 3.2. Chemicals and Reagents

All chemicals and reagents used in this study were of analytical or chromatographic grade and were used without further purification. Plant material extraction and preparation of crude extracts were carried out using ethanol and chloroform, produced by Sigma-Aldrich (St. Louis, MO, USA). Solvents for high-performance liquid chromatography (HPLC), including acetonitrile and acetic acid, were purchased from JT Baker (Phillipsburg, NJ, USA). Double-distilled water was used for the HPLC analysis. Reagents dedicated to liquid chromatography coupled with mass spectrometry (LC–MS), namely water, acetonitrile, and formic acid, were also obtained from JT Baker (USA, Phillipsburg, NJ, USA).

Solvents employed in the fractionation of raw extracts by CPC were sourced from Avantor Performance Materials (Gliwice, Poland) and were of reagent grade. Reagents used for the AChE fluorescence assay came from Sigma Aldrich (St. Louis, MO, USA) and included acetylcholinesterase from *Electrophorus electricus* (electric eel), Tris (hydroxymethyl) aminomethane (Tris), bovine serum albumin (BSA), 4-methylumbelliferyl acetate (4-MUA) and berberine chloride, a reference compound. Dimethyl sulfoxide (DMSO) and ethanol were purchased from POCH S.A. (Gliwice, Poland). Water was redistilled prior to use.

### 3.3. Extraction

Plant material was extracted using maceration and percolation techniques. Ethanol (96%), aqueous ethanol solutions (70% and 90%, prepared by dilution of 96% ethanol with distilled water to 1 L), and chloroform were used as extraction solvents. The ratio of plant material to solvent was 1:10 (*w*/*v*).

Dried and powdered aerial and underground parts of *A. salsa* were alkalized by moistening with a 30% potassium hydroxide (KOH) solution and then kept in a glass container for 1 h. The excess solution was then decanted, and the material was dried at room temperature.

For maceration, 0.05 kg of alkalized, air-dried, and powdered plant material was extracted five times with 70% and 90% aqueous ethanol and chloroform for 3 h at 55–60 °C (ethanolic solutions) and 40 °C (chloroform). The obtained extracts were combined, filtered, and centrifuged at 3500 rpm for 10 min. To obtain the aqueous extracts, the plant material (0.05 kg) was covered with water and kept in a boiling water bath for 15 min, then cooled for 45 min.

For percolation, 0.05 kg of non-alkalized, air-dried, and powdered plant material was extracted three times with 70% and 90% aqueous ethanol, with each extraction step preceded by 12 h of maceration. The extracts were combined and concentrated under reduced pressure using a rotary evaporator.

The concentrated extracts were transferred into pre-weighed flasks, dried to constant weight, and weighed. The obtained dry extracts were used for alkaloid isolation and stored at 4 °C until further analysis. A detailed list of the obtained extracts is provided in Table 3.

### 3.4. Compositional Studies of the Obtained Extracts by HPLC-ESI-QTOF-MS/MS

Approximately 10 mg of each extract was accurately weighed into Eppendorf tubes and dissolved in a methanol–water mixture (50:50, *v*/*v*) to obtain a final concentration of 10 mg/mL. The samples were vortex-mixed for 5 min to ensure complete dissolution. The resulting supernatant was filtered through a 0.1 µm nylon syringe filter directly into autosampler vials and subsequently subjected to HPLC–MS analysis. Comprehensive fingerprinting and untargeted metabolomic profiling were performed using an HPLC-ESI-QTOF-MS/MS system (Agilent Technologies, Santa Clara, CA, USA). The instrumentation comprised a 1200 Series HPLC system equipped with a vacuum degasser, autosampler, thermostated column compartment, and diode array detector (DAD), coupled to a G6530B quadrupole time-of-flight mass spectrometer fitted with a dual AJS electrospray ionisation (ESI) source. An additional isocratic pump was employed to introduce a reference mass solution. Chromatographic separation was achieved at a flow rate of 0.2 mL/min using an RP-18 Zorbax Eclipse Plus column (150 mm × 2.1 mm, 3.5 µm particle size). The mobile phase consisted of acetonitrile containing 0.1% formic acid and water with 0.1% formic acid, applied under the following gradient programme: 0 min—1% B; 10 min—20% B; 15 min—40% B; 17–22 min—95% B; 22.10–30 min—1% B. The reference mass solution was delivered isocratically at a flow rate of 0.01 mL/min.

Mass spectra were acquired following the injection of 10 µL of each sample into a freshly calibrated instrument. Analyses were conducted in both positive and negative ionisation modes, with three technical replicates per mode. The MS operating parameters were as follows: drying gas temperature 250 °C, sheath gas temperature 300 °C, gas flow rate 12 L/min, nebuliser pressure 35 psig, skimmer voltage 65 V, capillary voltage 3000 V, nozzle voltage 1000 V, fragmentor voltage 110 V, mass range *m*/*z* 40–1200 Da, and collision energies of 10 and 20 V. In each acquisition cycle, the two most intense precursor ions were selected for MS/MS fragmentation and subsequently excluded from further fragmentation for 0.2 min (dynamic exclusion).

Data acquisition and processing were carried out using MassHunter Workstation software (version B.10.00; Agilent Technologies). Putative compound identification was performed by comparison with publicly available mass spectral databases, including METLIN and Human Metabolome Database, as well as by reference to relevant scientific literature. The quantification of lupinine was performed by successive injections (n = 3) of the 1 and 0.1 mg/mL solutions of lupinine standard (>95%, (-)-Lupinine, Him Reakt, Moscow, Russian Federation) in the same method as the tested samples. Based on the collected peak areas, a direct comparison of the peak areas was performed to calculate the content of lupinine in the tested samples. 

The analysis of fractions’ composition and the selection of biphasic solvent systems were additionally supported by HPLC-DAD analyses. The injections were made on an Agilent 1100 system equipped with an autosampler, column thermostat, and diode array detector (DAD). The separation was carried out on a Zorbax Eclipse XDB-C18 column (4.6 × 250 mm, 5 μm particle size). The mobile phase consisted of methanol and water acidified with 1% acetic acid (*v*/*v*), and gradient elution was applied as described above. The flow rate was maintained at 1 mL/min, and the column temperature was set at 25 °C. The injection volume was 10 μL, and each sample was injected in triplicate. Detection was performed at four wavelengths: 254, 280, 320, and 365 nm. The total analysis time was 50 min. The obtained chromatograms were used for comparative analysis of extract composition and for evaluation of compound distribution in different solvent systems.

### 3.5. The Fractionation of the Ethanol Extract by Centrifugal Partition Chromatography

The fractionation of the 90% ethanol extract was performed using a centrifugal partition chromatograph under optimised conditions, using a biphasic solvent system composed of methyl tert-butyl ether (MtBE), acetonitrile (ACN), n-butanol (BuOH), and water in the ratio of 2:2:1:5 (*v*/*v*/*v*/*v*). For the separation, the solvent system was prepared in a separatory funnel and allowed to settle until complete phase separation was achieved. The upper and lower phases were subsequently separated and degassed prior to chromatographic use. The separation was carried out on a Spot CPC system (Armen Instrument, Saint-Avé, France) equipped with a 250 mL CPC rotor, a UV–Vis detector, and an automated fraction collector. The chromatographic run was conducted in the ascending mode. Initially, the column was entirely filled with the stationary phase, after which the mobile phase was pumped through the system together with the injected sample. UV detection was performed simultaneously at 201 and 254 nm. The elution step was conducted from 1 to 50 min, followed by extrusion of the stationary phase between 50 and 120 min to ensure complete recovery of retained constituents. In the course of the analysis, fractions of 8 mL were collected throughout. From each collected fraction, 2 mL aliquots were transferred to Eppendorf vials, evaporated to dryness using an Eppendorf Concentrator Plus evaporator (Hamburg, Germany) under reduced pressure, weighed and re-dissolved in methanol at a concentration of 10 mg/mL. The fractions were later filtered through 0.1 µm nylon syringe filters into glass vials and used for compositional analysis in the HPLC-MS in the method described above.

### 3.6. Fluorometric AChE Inhibition Assay

AChE potential was measured using a Greiner CELLSTAR^®^ 96-well black plate (Greiner Bio-One, Monore, NC, USA). The results were obtained with the help of Promega GloMax^®^ Explorer Microplate Reader (Promega Corporation, Madison, WI, USA) in fluorescence mode. Studied samples (fractions) were assayed in triplicate. Fractions and studied extracts were tested at a concentration of 10 mg/mL, whereas berberine chloride was tested at 1 mg/mL. Berberine chloride was used as a reference inhibitor, while dimethyl sulfoxide (DMSO) was used as a solvent control. The enzyme was added to the applied samples. After incubating at 37 °C for 25 min, the substrate 4-methylumbelliferyl acetate (4-MUA) was added. The fluorescence of the samples was measured after 20 min of re-incubation at 365 nm. The obtained results were processed using Microsoft Excel.

## 4. Conclusions

The present study demonstrates that *Anabasis salsa* is a rich source of biologically active metabolites, including alkaloids, phenolic acids, and flavonoids. The application of HPLC-ESI-QTOF-MS/MS enabled comprehensive profiling of the extract, confirming the presence of key alkaloids such as anabasine and lupinine.

Centrifugal partition chromatography (CPC) proved to be an efficient and selective technique for the fractionation of complex plant extracts. The optimised biphasic solvent system allowed for effective separation and enabled the isolation of lupinine in a purified fraction without the use of solid stationary phases.

Biological evaluation of CPC fractions revealed significant acetylcholinesterase (AChE) inhibitory activity, with the most active fractions containing lupinine. The isolated compound exhibited slightly stronger inhibitory activity than berberine under the applied experimental conditions, highlighting its relevance as a potent natural AChE inhibitor.

These findings indicate that lupinine is a significant bioactive constituent and a major contributor to the acetylcholinesterase inhibitory activity observed in *A. salsa* extracts. Given the established role of acetylcholinesterase inhibition in the symptomatic treatment of neurodegenerative disorders, lupinine may represent a promising lead compound for further studies on neuroactive agents. However, its neuroprotective activity was not directly evaluated in the present study and requires further investigation. Moreover, the study confirms that CPC is a powerful and scalable approach for the isolation of alkaloids from complex plant matrices, offering advantages in terms of efficiency, selectivity, and reduced solvent consumption.

## Figures and Tables

**Table 1:** The tentatively identified alkaloids in the extracts from *Anabasis salsa* (the compounds in bold highlight the alkaloids identified based on the comparison with the standard compounds).

No	Ion (+/−)	Rt (min)	Neutral Molecular Formula	*m/z* Calculated	*m/z* Experimental	MS/MS Fragments	DBE	Error [ppm]	Proposed Compound	Extract
1	+	2.837	C_10_H_14_N_2_	163.1230	163.1233	146.0959 134.0959 120.0805	5	−2.01	**Anabasin**	N9–N11. N13–N14
2	+	2.93	C_10_H_19_NO	170.1539	170.1540	152.1438 137.1201 124.1127	2	−0.35	**Lupinine**	N9 N12–N15
3	+	12.818	C_18_H_20_N_2_O_2_	297.1598	297.1600	255.1084 184.0716	10	−0.83	Lupinine analogue, quinolizidine alkaloid	N9–N14, N16, N18–19, N23–N24
4	+	13.766	C_18_H_20_N_2_O_3_	313.1547	313.1546	253.0846 150.0920	10	0,22	N-oxide/hydroxylated quinolizidine alkaloid (e.g., N-oxide of lupinine)	N13, N14, N16, N18,
5	+	13.216	C_14_H_17_NO_2_	232.1332	232.1333	188.0595 118.0555	7	−0.41	Pyridine alkaloid derivative	
6	+	4.75	C_10_H_15_NO	166.1226	166.1223	121.0646 103.0540 93.0697	2.06	4	Jaksartinine	N9–N11, N17–N19
7	−	2.071	C_7_H_12_O_6_	191.0561	191.0555	173.0454 173.0454 111.0088 85.0301	2	3.18	Quinic acid	N11–N13 N15–N16
8	−	2.98	C_6_H_8_O_7_	191.0197	191.0206	173.0078 111.0096 85.0308	3	−4.55	Citric acid	N10
9	−	2.805	C_4_H_6_O_5_	133.0142	133.0142	115.0037	2	−1.89	Malic acid	N13–N16
10	−	3.822	C_4_H_6_O_4_	117.0193	117.0193	99.0195 73.0306	2	−0.57	Succinic acid	N13 N14 N15 N16
11	−	11.839	C_8_H_7_O_4_	167.0351	167.0348	137.0243 123.0442 109.0292	5	−9.63	Vanillic acid	N13
12	−	11.894	C_14_H_20_O_8_	315.1085	315.1072	153.0550 123.0443	5	4.24	Protocatechuic acid hexoside isomer	N10–N13 N16
13	−	13.224	C_21_H_20_O_11_	447.0933	447.1508	293.0879 285.0975 153.0548 123.0448	6	1.31	Luteolin glucoside (orientin)	N11 N13
14	−	13.278	C_15_H_20_O_10_	359.0984	359.0998	197.0444 179.0550 166.9981 153.0549	6	−3.97	Syringic acid hexoside	N10–N14 N16
15	−	13.445	C_13_H_16_O_9_	315.0722	315.0738	153.0186 109.0292	6	−5.2	Protocatechuic acid hexoside isomer	N10–N14
16	−	13.562	C_13_H_16_O_10_	331.0671	331.0683	168.0059 125.0235	6	−3.7	Galloylglucose	N10–N11 N13 N16
17	−	14.579	C_13_H_16_O_8_	299.0772	299.0775	137.0237 93.0342	6	−0.86	Hydroxybenzoic acid glucoside	N10–N11 N13 N16
18	−	14.796 20.963	C_7_H_6_O_3_	137.0244	137.0244	93.0343	5	0.13	Hydroxybenzoic acid	N10–N14 N16
19		15.030	C_15_H_1_8O_10_	357.0827	357.1022	153.0551 135.0448 59.0147	7	0.06	Caffeoylgluconic acid isomer	N10
20	−	15.146	C_7_H_6_O_3_	137.0244	137.0240	93.0345	5	3.03	Hydroxybenzoic acid	N14
21	−	15.125	C_16_H_22_O_10_	373.114	373.1158	329.1244 165.0548 89.0255	6	−4.76	Swertiamarin	N10–N13 N15 N16
22	−	15.48	C_12_H_14_O_9_	301.0565	301.0568	168.0054	6	−0.97	Pyrogallol glucuronide	N13 N15
23		15.630	C_17_H_22_O_9_	367.1107	367.1040	193.0496 173.0444 134.0368 85.0285	8	−1.48	Feruloylquinic acid	N13 N15
24	−	15.730	C_7_H_6_O_4_	153.0193	153.0191	109.0287	5	1.51	Protocatechuic acid	N10–N14 N16
25	−	16.893	C_27_H_30_O_15_	593.1512	593.1505	473.1057 353.0673 153.0187	13	1.17	Quercetin dirhamnoside	N11
26	−	16.576	C_9_H_8_O_4_	179.035	179.0351	135.0446	6	−0.1	Caffeic acid	N13 N15
27	−	17.071	C_9_H_10_O_5_	197.0455	197.0453	135.0841	5	1.25	Syringic acid	N10
28	−	17.281	C_16_H_18_O_8_	337.0929	337.0930	191.0548 173.0443 163.0383 135.0444	8	−0.32	Coumaroylquinic acid	N13 N15
29	−	17.748	C_14_H_18_O_9_	329.0878	329.0883	167.0352	6	−1.5	Vanillic acid hexoside	N10 N13 N16
30	−	17.844	C_16_H_20_O_10_	371.0984	371.1001	311.1083 249.0605 175.0240 121.0285	7	−4.65	Dihydroferulic acid glucuronide	N11 N13 N16
31	−	18.632	C_9_H_78_O_3_	163.0401	163.0400	119.0497	6	0.41	Coumaric acid	N10 N12
32	−	19.299	C_27_H_30_O_16_	609.1461	609.1431	301.0339	13	4.93	(Rutoside) Quercetin-rutinoside	N10
33	−	19.311	C_10_H_10_O_4_	193.0506	193.0508	178.0265 136.0159	6	−0.86	Ferulic acids	N12
34		19.950	C_22_H_22_O_12_	477.1038	477.1035	315.0482 301.0341	12	0.73	Isorhamnetin hexoside	N10
35	−	21.201	C_15_H_18_O_9_	341.0878	341.0907	180.9830 96.9605	7	−10.51	Caffeic acid hexoside	N10–N13 N16
36	−	22.168	C_7_H_6_O_4_	153.0193	153.0191	135.0079 109.0289	5	1.51	Gentisic acids	N14

(Ion—ionisation mode, error—error of *m*/*z* measurement, DBE—double bond equivalent value, MS/MS—registered MS/MS fragments).

**Table 2:** The averaged percentage content of lupinine in the tested extracts (green—the highest value, red—the lowest value).

Extract No	9	10	11	12	13	14	15	16
Extract name	AS-70P	AS-70t	AS-90P	AS-90tP	AS-90t	AS-K-90	AS-X-t	AS-K-x
Lupinine content [%]	0.0727	0.0033	0.0202	6.3523	0.5554	0.0265	0.0179	0.0080
RSD [%]	2.57	1.39	2.66	1.40	1.21	2.99	0.816	4.68

**Table 3:** The list of analysed extracts of *A. salsa* in this study.

No.	Extract Name	Solvent and Concentration	Extraction Method	Temperature	Length	Plant Organ
9	AS-70P	70% ethanol	percolation	RT	12 h	aerial parts
10	AS-70t	70% ethanol	maceration	55–60 °C	3 h	aerial parts
11	AS-90P	90% ethanol	percolation	RT	12 h	aerial parts
12	AS-90tP	90% ethanol	maceration (alkalized plant material)	55–60 °C	3 h	aerial parts
13	AS-90t	90% ethanol	maceration	55–60 °C	3 h	aerial parts
14	AS-K-90	90% ethanol	maceration	55–60 °C	3 h	roots
15	AS-X-t	chloroform	maceration	40 °C	3 h	aerial parts
16	AS-K-x	chloroform	maceration	40 °C	3 h	roots
17	AS-water K	water	maceration	RT	3 h	roots
18	AS-water H	water	maceration	RT	3 h	aerial parts

## Data Availability

The original contributions presented in this study are included in the article/Supplementary Material. Further inquiries can be directed to the corresponding authors.

## Supplementary Materials

The following supporting information can be downloaded at: https://www.mdpi.com/article/10.3390/molecules31142452/s1, Table S1: The MS/MS spectra recorded for the tentatively identified compounds. Table S2: The list of biphasic solvent systems that were prepared for the evaluation of the partition coefficient values, with system 4 as the most beneficial one for the assessed extract. Table S3: The results of the K value assessment for the six evaluated biphasic solvent systems. Table S4: The HPLC chromatograms of the most active fractions: 17–18 from CPC (4), and fraction 24 (9). Figure S1: The MS and MS/MS spectra of the compound from fraction 17–18 from CPC (4). Figure S2: The structures of lupinine and anabasin.
