# Supplementary material for: Lupinine as an Acetylcholinesterase Inhibitor from Anabasis salsa (C.A. Mey.) Benth. ex Volkens—Isolation by Centrifugal Partition Chromatography
Source: Molecules. 2026 Jul 13;31(14):2452. doi: 10.3390/molecules31142452 (PMC13415605; doi:10.3390/molecules31142452)

**SUPPLEMENTARY FILE**

# Lupinine as an acetylcholinesterase inhibitor from *Anabasis salsa* (C.A. Mey.) Benth. ex Volkens – quantitative analysis and isolation by centrifugal partition chromatography

Pernesh Zh. Bekisheva [1], Malgorzata Kozyra [2], Maryna Koval [2,3], Khorlan I. Itzhanova [1], Zhangeldy S. Nurmaganbetov [1,*], Wirginia Kukula-Koch [2,*]

Table S1. The MS/MS spectra recorded for the tentatively identified compounds.

<table>
<tr><td>Quinic acid</td><td>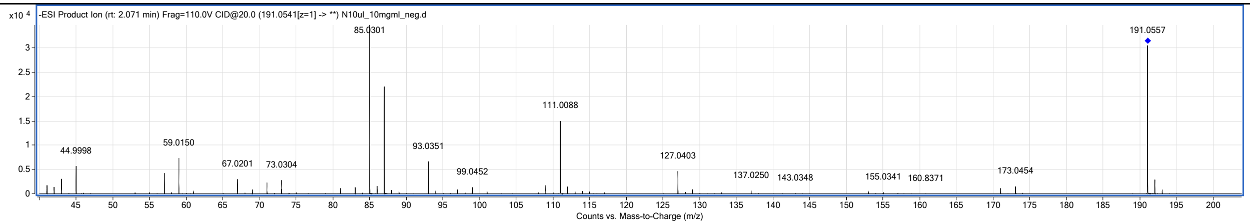
</td></tr>
<tr><td>Anabasine</td><td>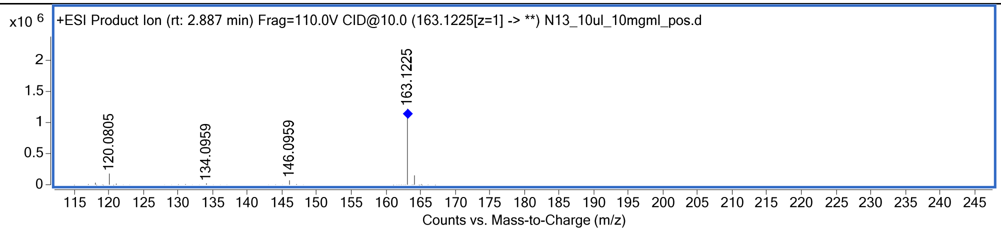
</td></tr>
</table>

<table>
<tr><td>Lupinine</td><td>
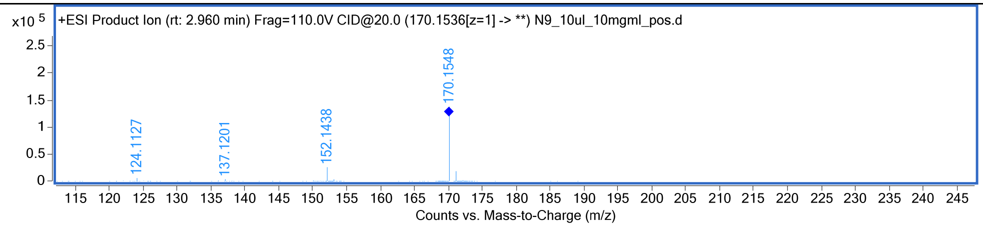

</td></tr>
<tr><td>Citric acid</td><td>
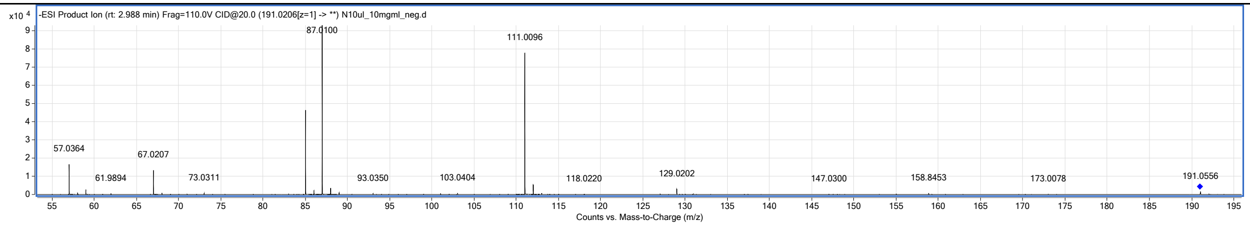

</td></tr>
<tr><td>Malic acid</td><td>
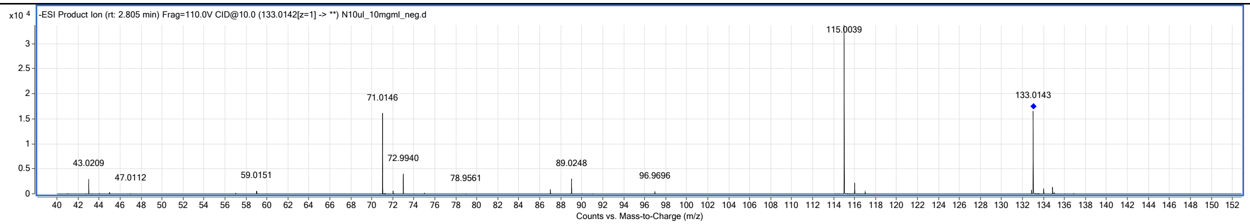

</td></tr>
<tr><td>Succinic acid</td><td>
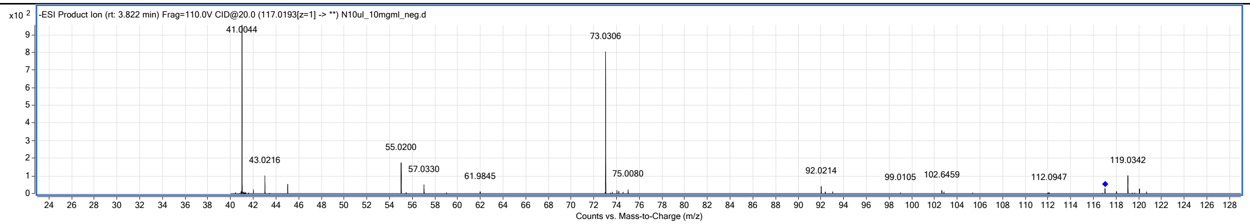

</td></tr>
<tr><td>Vanilic acid</td><td>
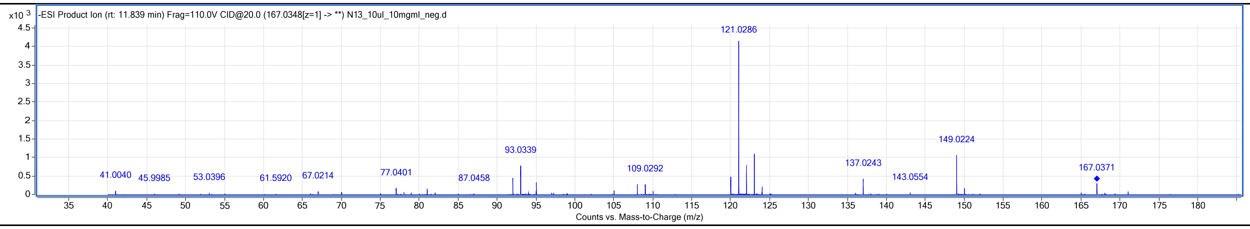

</td></tr>
</table>

| | |
|---|---|
| Protocatechuic acid -O -hexoside isomer | 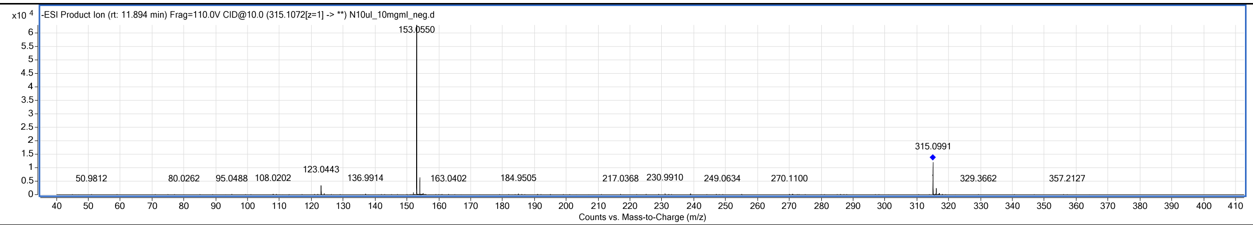 |
| Luteolin 8-O-glucoside (orierntin) | 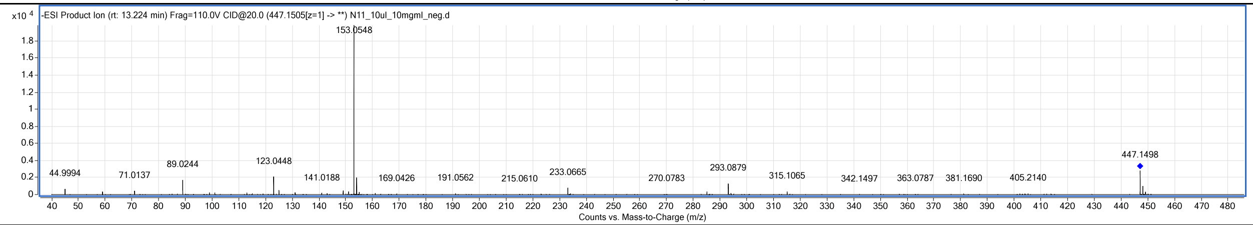 |
| Syringic acid 4-O-hexoside | 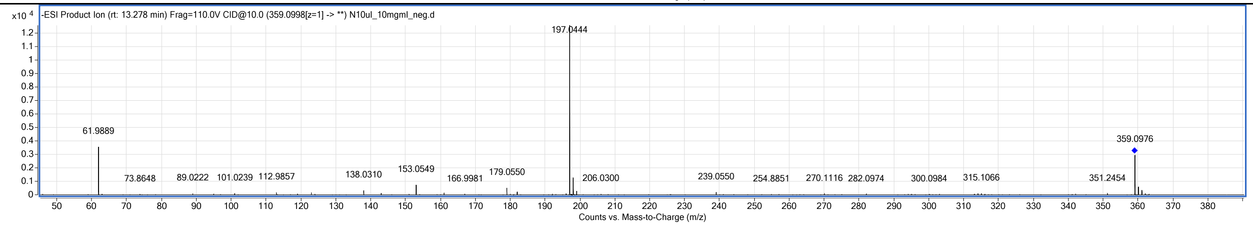 |
| Protocatechuic acid -O-hexoside isomer | 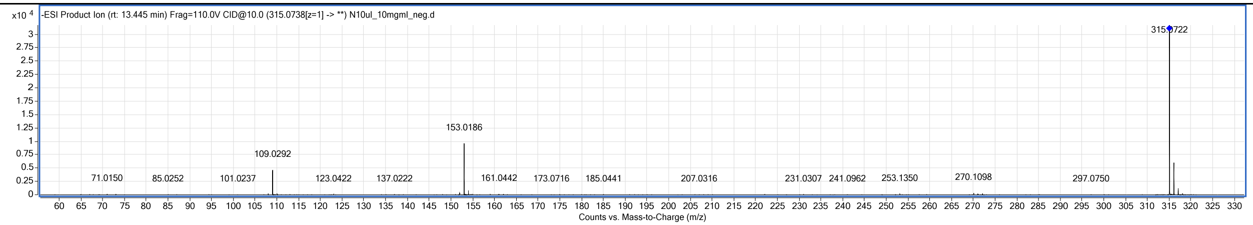 |
| 1-O-Galloylglucose | 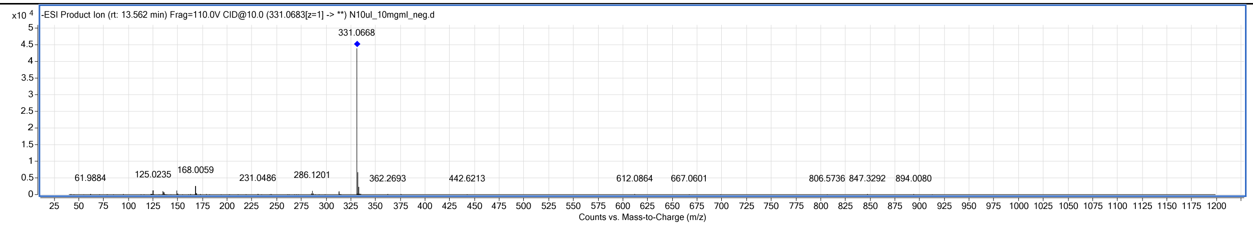 |

4-hydroxybenzoic acid

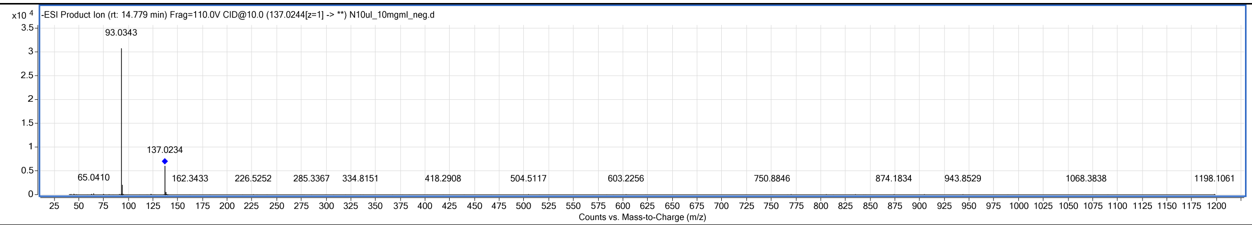


Caffeoylgluconic acid isomer

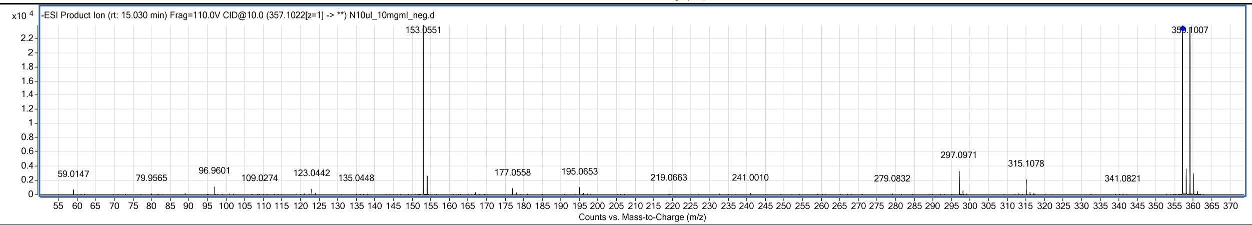


Swertiamarin

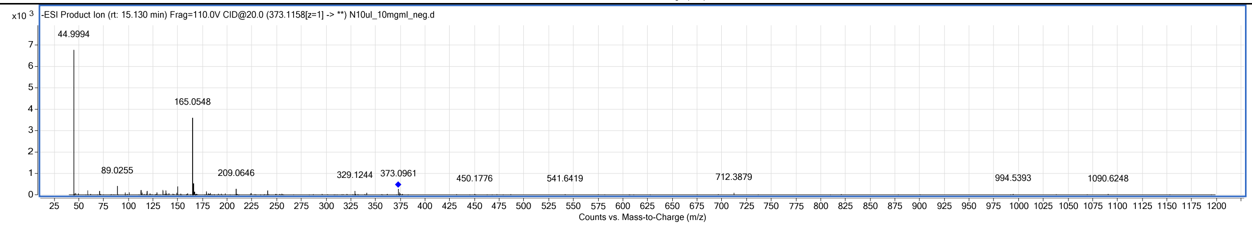


Feruloquinic acid

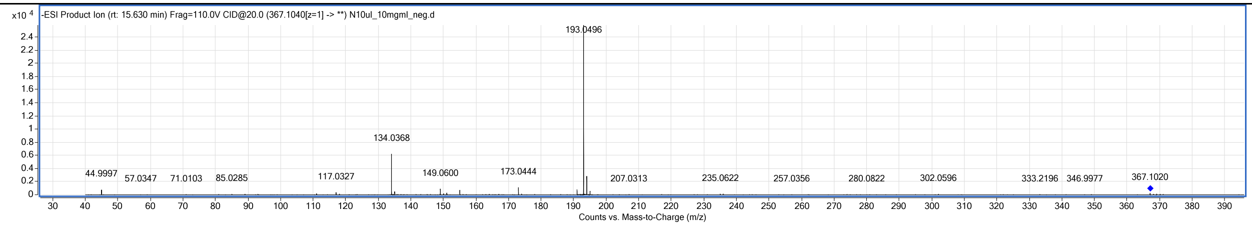


Protocatechuic acid

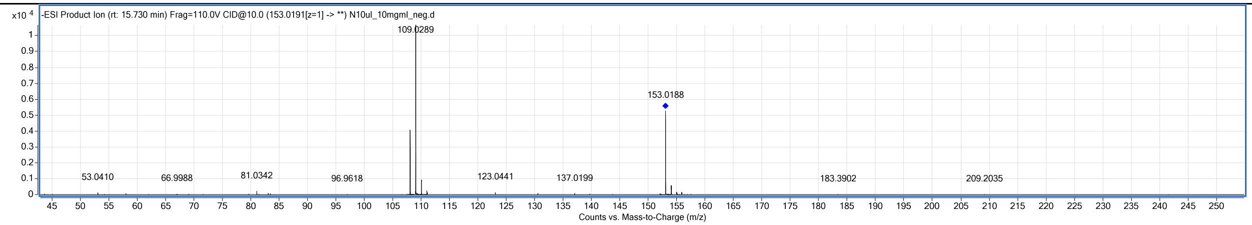

| | |
|---|---|
| Quercetin-dirhamnoside | 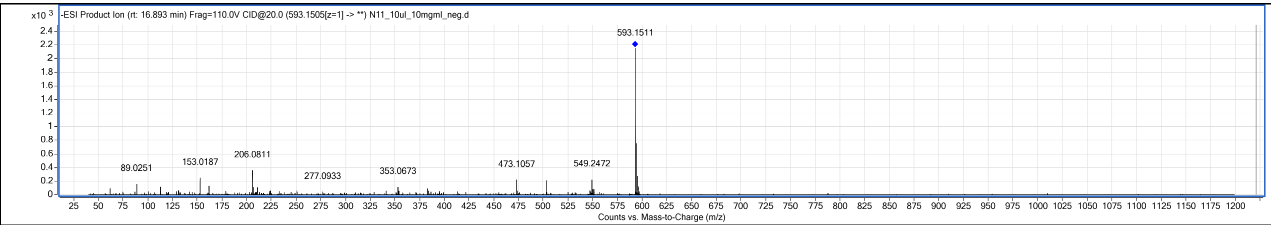 |
| Caffeic acid | 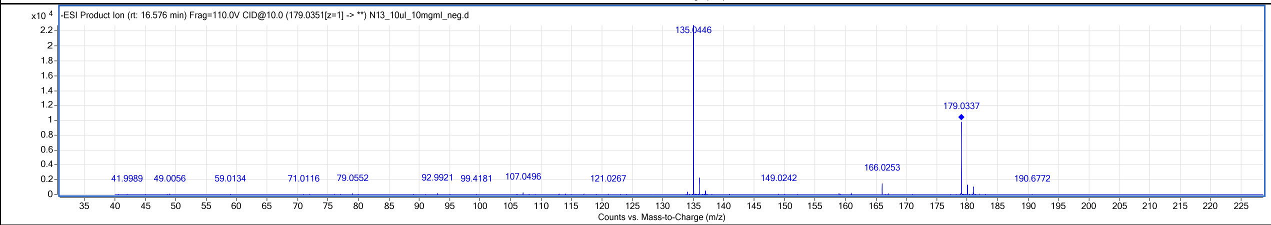 |
| Syringic acid | 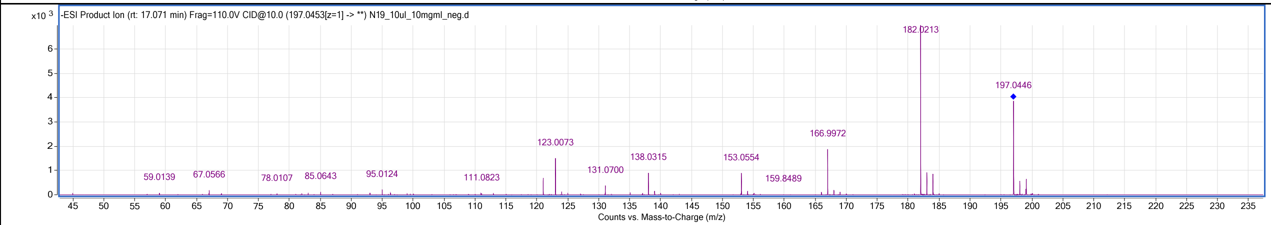 |
| Vanilic acid 4-O-hexoside | 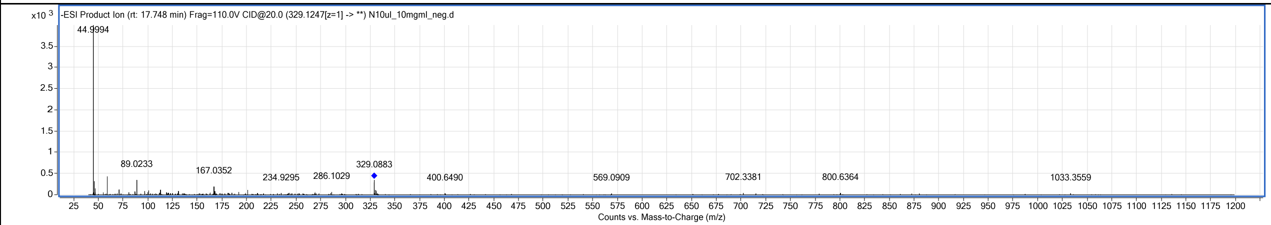 |
| Dihydroferulic acid 4-O-glucuronide | 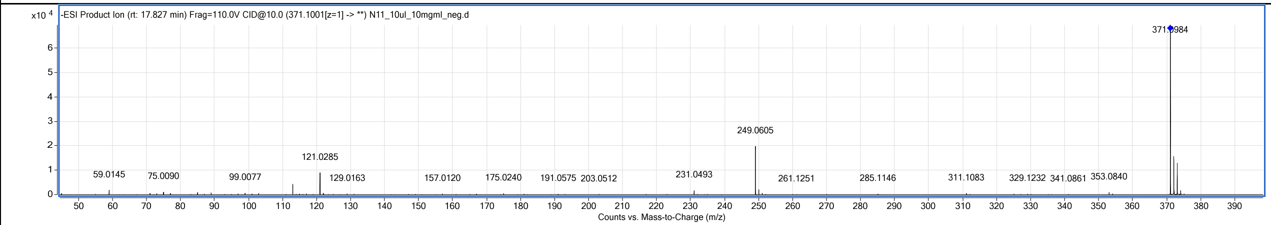 |

| | |
|---|---|
| p-coumaric acid | 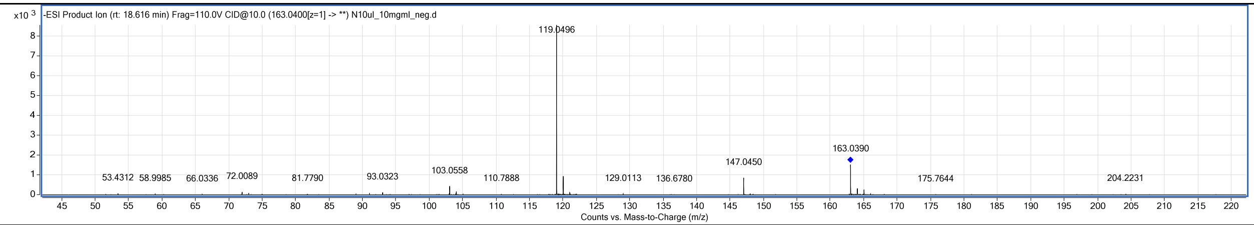 |
| (RUTIN) Quercetin-3-rutinoside | 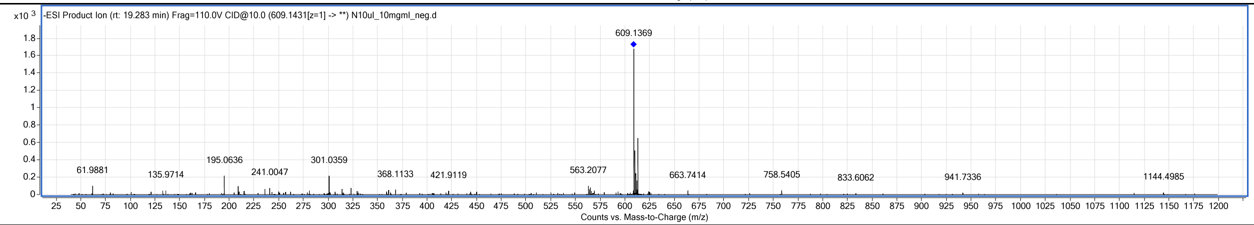 |
| Ferulic acids | 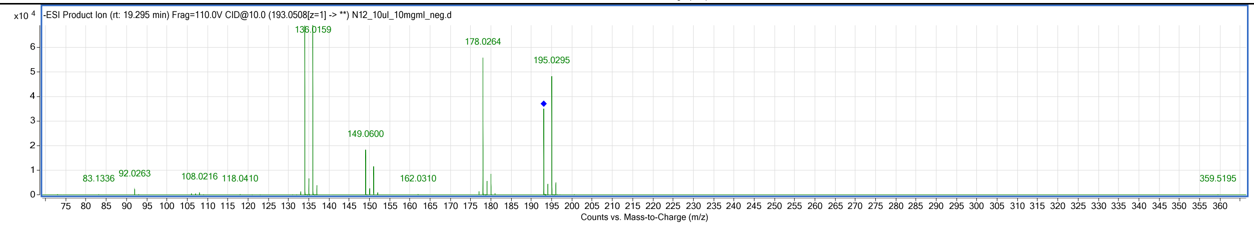 |
| Isorhamnetin-O-hexoside | 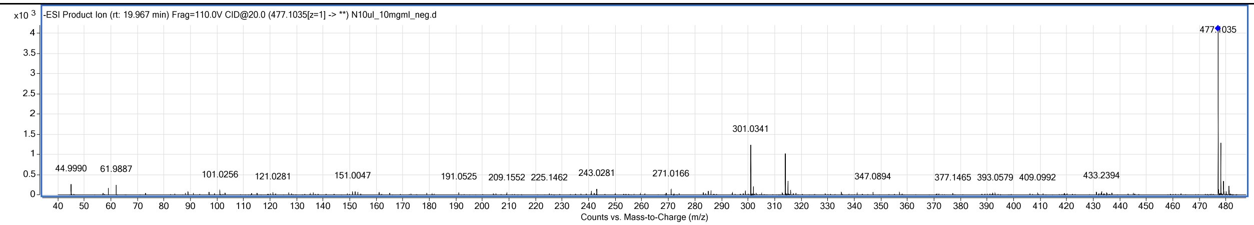 |
| Caffeic acid hexoside | 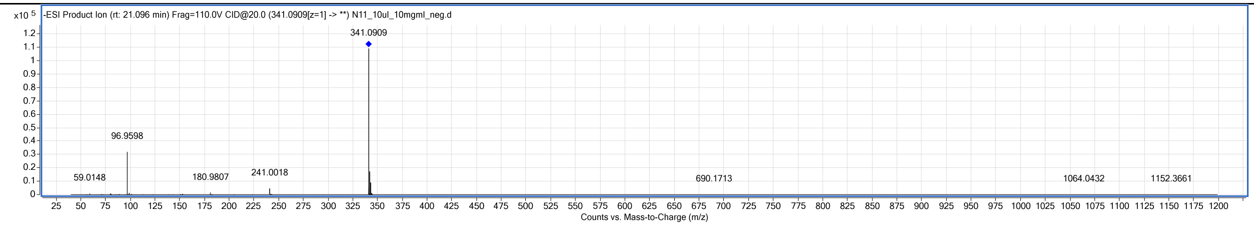 |
| Lupinine ana-logue, quinoliz-idine alkaloid | 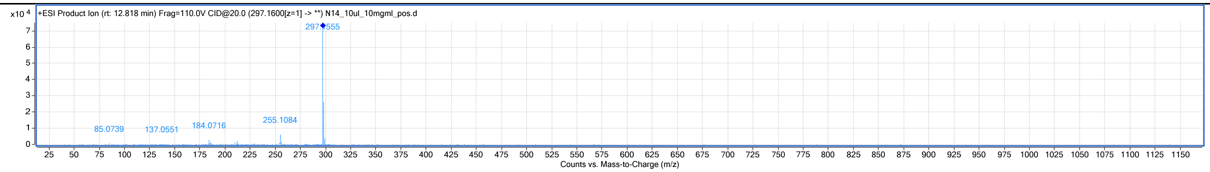 |

| | |
|---|---|
| N-oxide/hydroxylated quino-lizidine alkaloid (e.g. N-oxide of lupinine) | 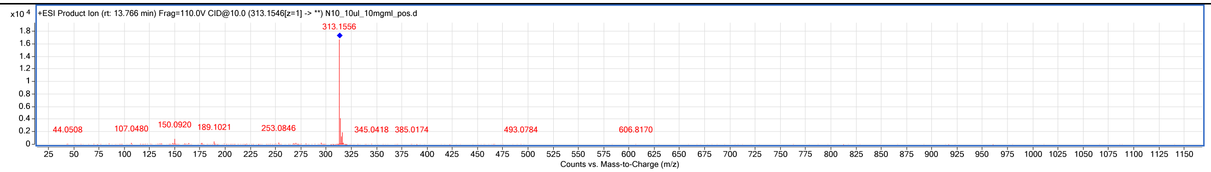 |
| Pyridine alkaloid derivative. | 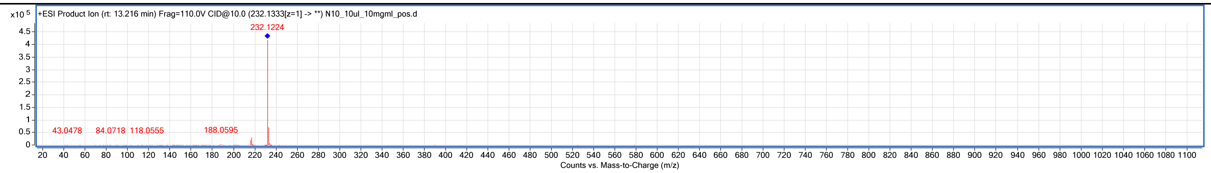 |
| yaksartinine | 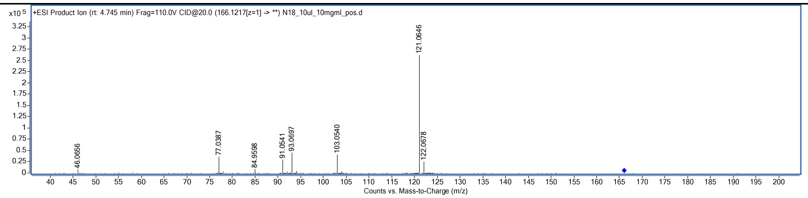 |
| Gentisic acid | 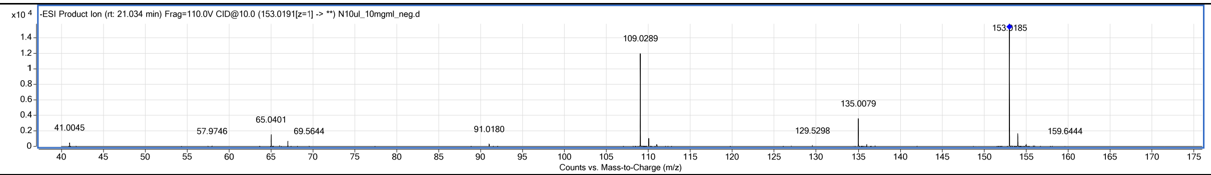 |
| Hydroxybenzoic acid | 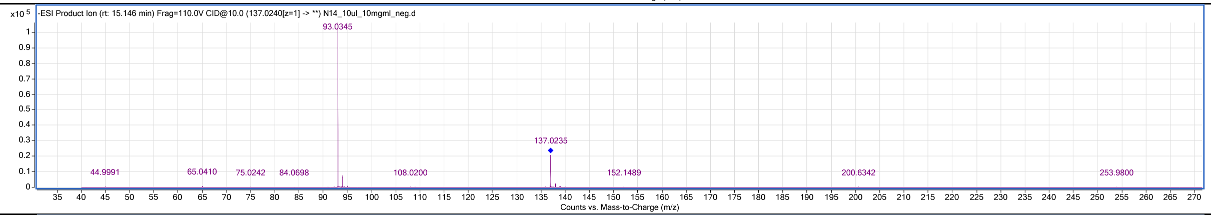 |
| Pyrogallol glucuronide | 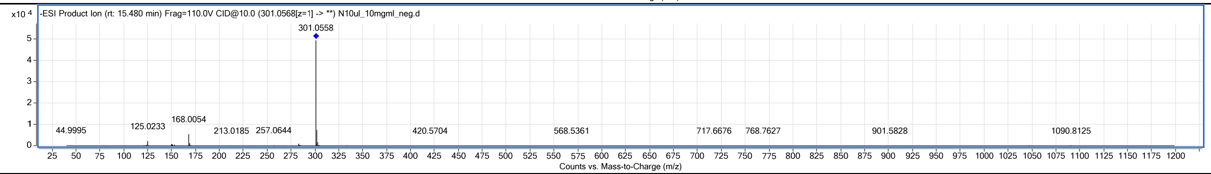 |

<table>
<tr>
<td>Coumaroyl-quinic acid</td>
<td>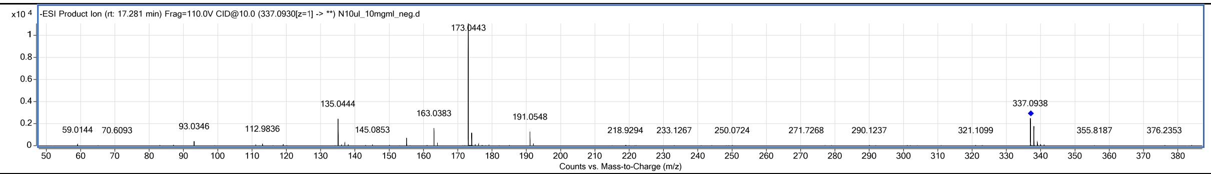
</td>
</tr>
<tr>
<td>Hydroxybenzoic acid glucoside</td>
<td>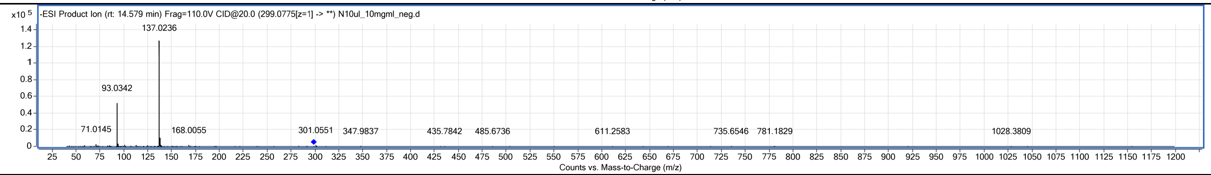
</td>
</tr>
</table>

**Table S2**. The list of biphasic solvent systems that were prepared for the evaluation of the partition coefficient values with the system 4 as the most beneficial one for the assessed extract.

| System No. | Solvent system composition | Ratio (v/v) | Additives |
|---|---|---|---|
| **1** | MTBE : $H_2O$ | 1 : 1 | 10 mM TEA, 10 mM HCl |
| **2** | n-Hexane : n-BuOH : EtOH : $H_2O$ | 3 : 12 : 6 : 15 | — |
| **3** | n-Hexane : n-BuOH : EtOH : $H_2O$ | 1 : 14 : 6 : 15 | — |
| **4** | **MTBE : ACN : n-BuOH : $H_2O$** | **2 : 2 : 1 : 5** | — |
| **5** | MTBE : n-BuOH : ACN : 10 mM HCl | 2 : 2 : 1 : 5 | 10 mM HCl |
| **6** | n-Hexane : EtOAc : EtOH : $H_2O$ | 5 : 3 : 4 : 4 | 10 mM HCl |

**Table S3**. The results of the K value assessment for the six evaluated biphasic solvent systems

<table>
<tr><td>Biphasic solvent No 1</td><td>Biphasic solvent No 4</td></tr>
<tr><td>1в
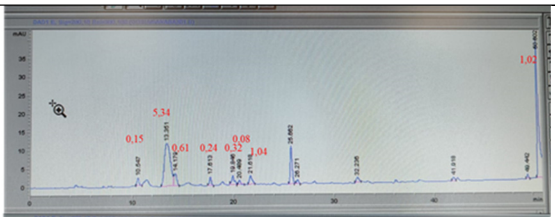

2н
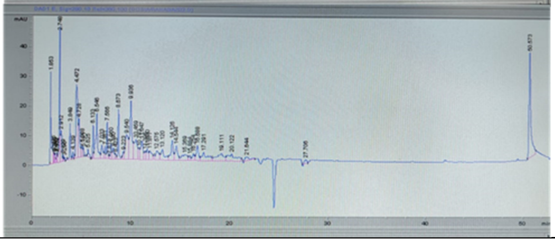</td><td>7 в
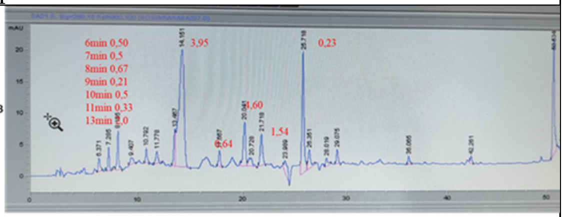

8 н
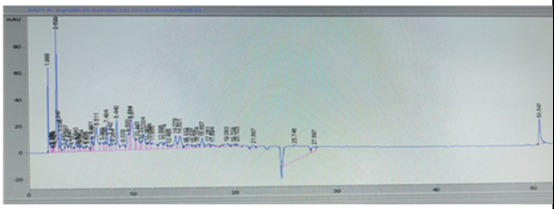</td></tr>
<tr><td>Biphasic solvent No 2</td><td>Biphasic solvent No 5</td></tr>
<tr><td>3 в
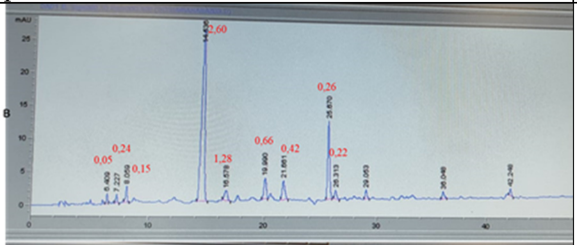

4 н
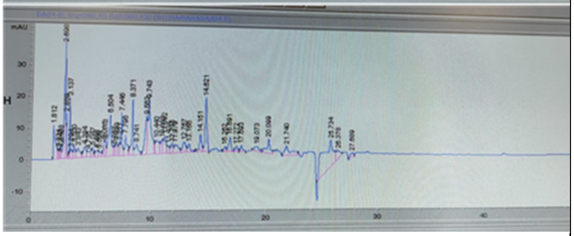</td><td>9 в
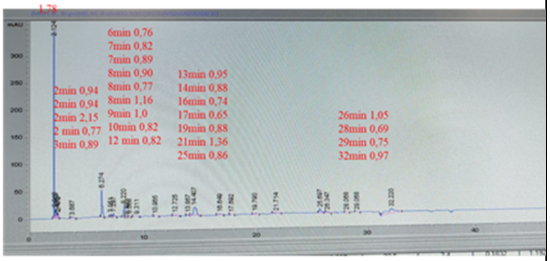

10 н
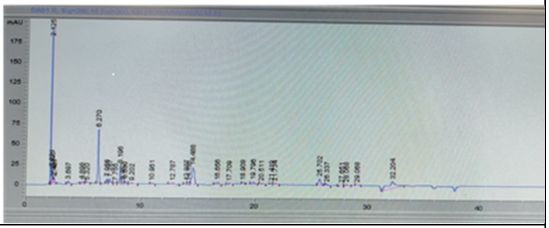</td></tr>
<tr><td>Biphasic solvent No 3</td><td>Biphasic solvent No 6</td></tr>
<tr><td>5 в
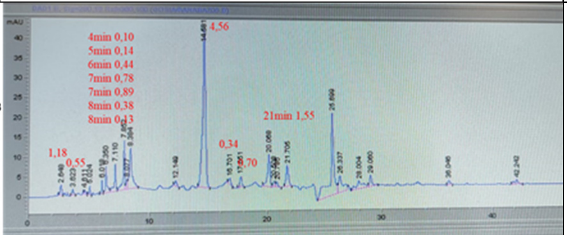

6 н
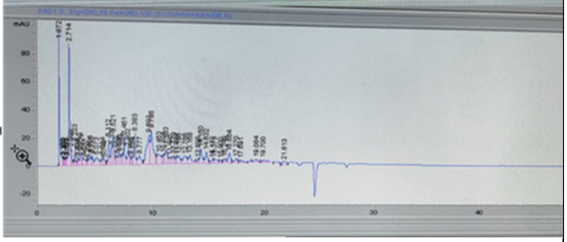</td><td>11 в
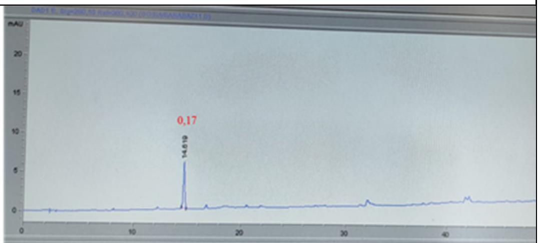

12н
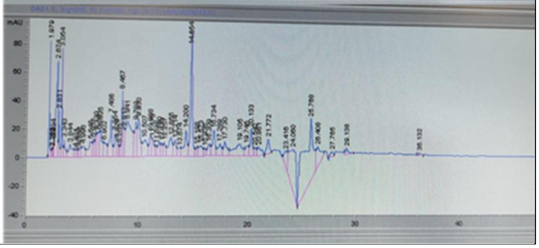</td></tr>
</table>

Table S4. The HPLC chromatograms of the most active fractions: 17-18 from CPC (4), and fraction 24 (9).

| Fraction 17-18 from CPC ( 4) |
| --- |

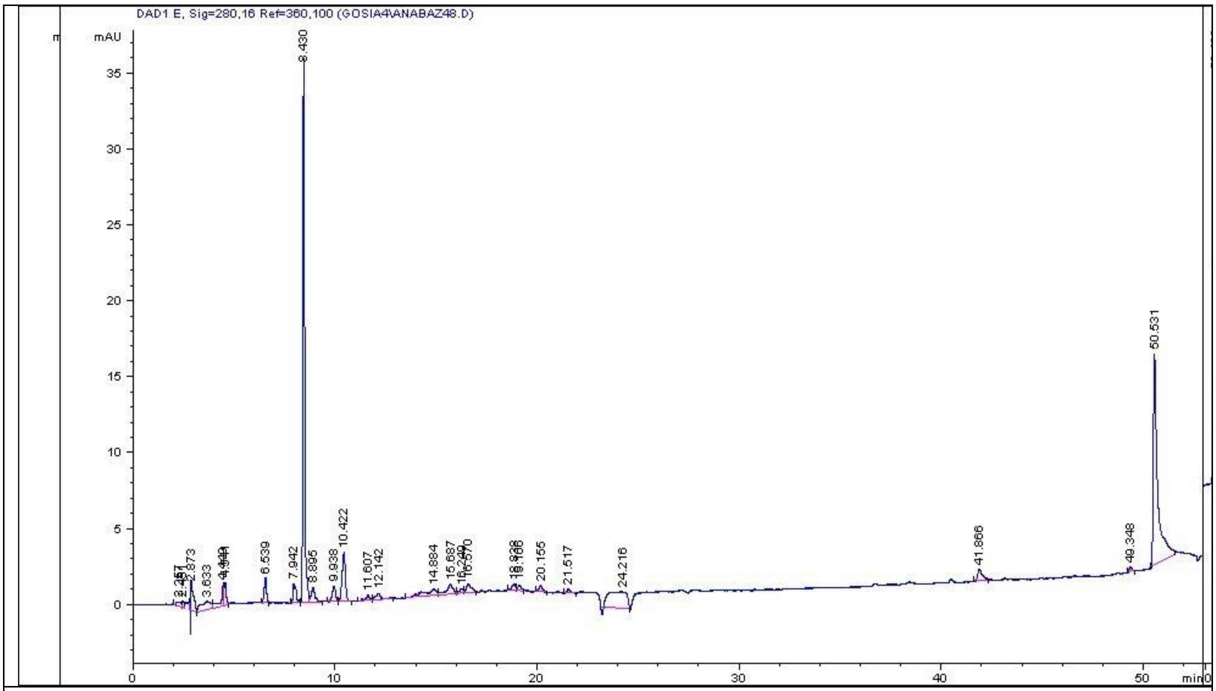


Fraction 24 from CPC (9)

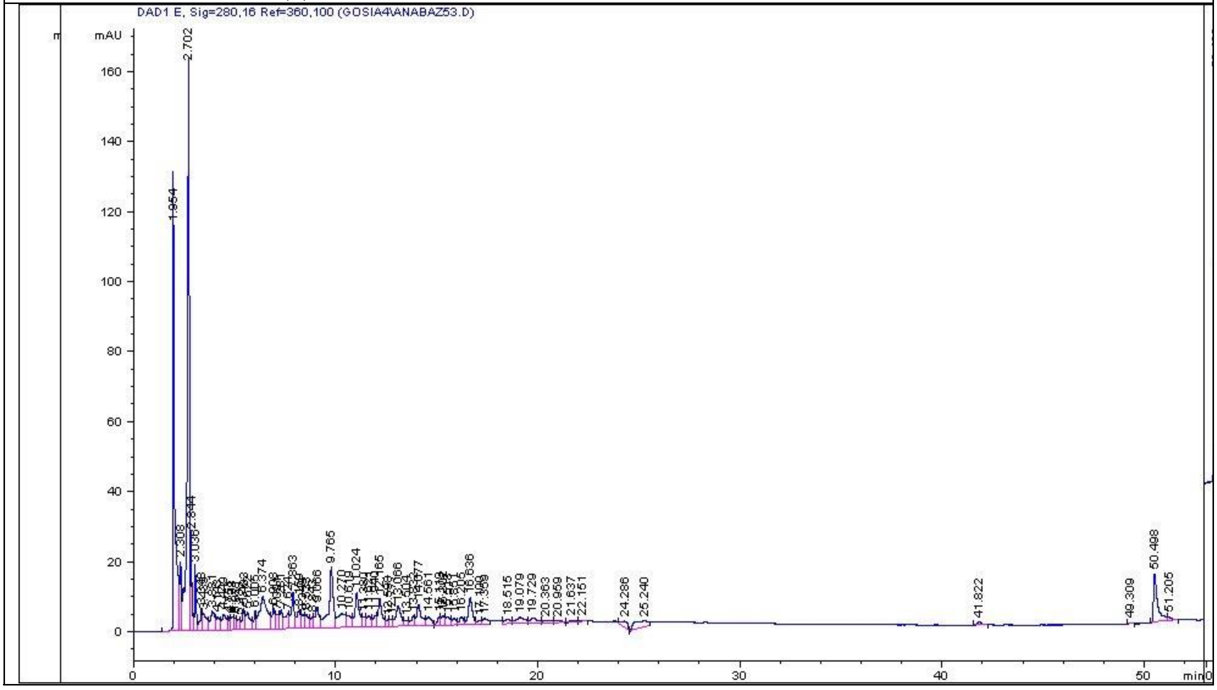

**Figure S1**. The MS and MS/MS spectra of the compound from fraction 17-18 from CPC (4)

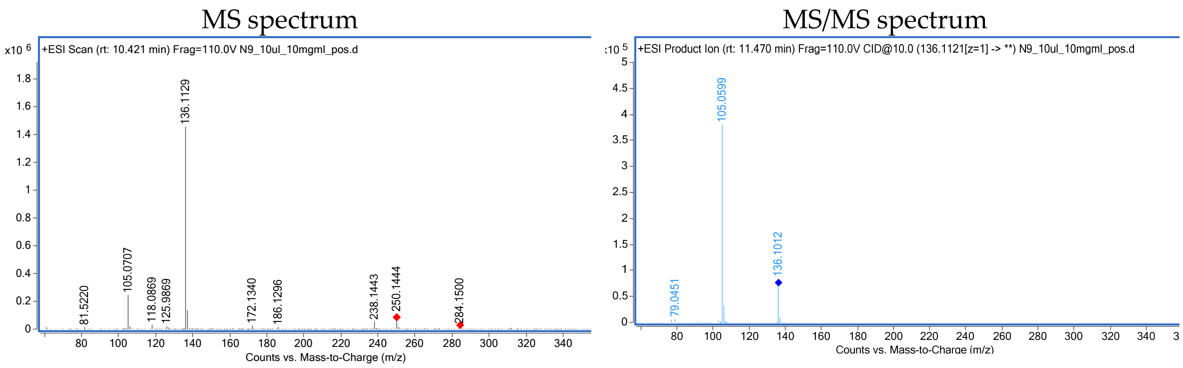


**Figure S2**. The structures of lupinine and anabasin.

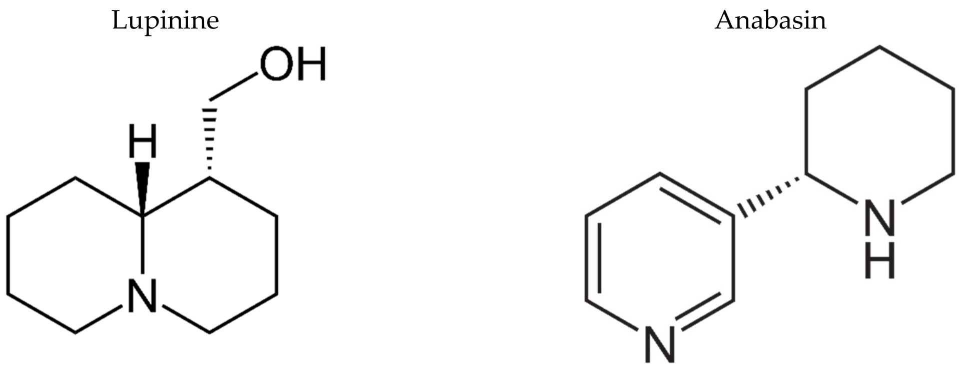

Supplement: Supplementary file 1 [file molecules-31-02452-s001.zip › molecules-4382996-supplementary.pdf]
